# Efficacy of artemether–lumefantrine and dihydroartemisinin–piperaquine for the treatment of uncomplicated malaria in Papua New Guinea

**DOI:** 10.1186/s12936-018-2494-z

**Published:** 2018-10-05

**Authors:** Livingstone Tavul, Manuel W. Hetzel, Albina Teliki, Dorish Walsh, Benson Kiniboro, Lawrence Rare, Justin Pulford, Peter M. Siba, Stephan Karl, Leo Makita, Leanne Robinson, Johanna H. Kattenberg, Moses Laman, Gilchrist Oswyn, Ivo Mueller

**Affiliations:** 1 Papua New Guinea Institute of Medical Research, PO Box 378, Madang, Papua New Guinea; 2 Swiss Tropical and Public Health Institute, PO Box, 4002, Basel, Switzerland; 3 University of Basel, Petersplatz 1, 4003, Basel, Switzerland; 4 Liverpool School of Tropical Medicine, Pembroke Place, Liverpool, L35QA UK; 5 Infection and Immunity Division, Walter and Eliza Hall Institute, Melbourne, Australia; 6 Department of Medical Biology, University of Melbourne, Melbourne, Australia; 7 National Department of Health, PO Box 807, Waigani, NCD Papua New Guinea; 8 Milne Bay Provincial Health Authority, Lock Bag 402, Alotau, Papua New Guinea; 9 Institut Pasteur, 25-28, rue du Docteur-Roux, Cedex 15, 75724 Paris, France; 10 Institute of Tropical Medicine in Antwerp, Kronenburgstraat 43, 2000 Antwerp, Belgium

**Keywords:** Efficacy, Artemether–lumefantrine, Dihydroartemisinin–piperaquine, *Plasmodium falciparum*, *Plasmodium vivax*, Malaria, In vivo, In vitro

## Abstract

**Background:**

In 2009, the Papua New Guinea (PNG) Department of Health adopted artemether–lumefantrine (AL) and dihydroartemisinin–piperaquine (DHA-PPQ) as the first- and second-line treatments for uncomplicated malaria, respectively. This study was conducted to assess the efficacy of both drugs following adoption of the new policy.

**Methods:**

Between June 2012 and September 2014, a therapeutic efficacy study was conducted in East Sepik and Milne Bay Provinces of PNG in accordance with the standard World Health Organization (WHO) protocol for surveillance of anti-malarial drug efficacy. Patients ≥ 6 months of age with microscopy confirmed *Plasmodium falciparum* or *Plasmodium vivax* mono-infections were enrolled, treated with AL or DHA-PPQ, and followed up for 42 days. Study endpoints were adequate clinical and parasitological response (ACPR) on days 28 and 42. The in vitro efficacy of anti-malarials and the prevalence of selected molecular markers of resistance were also determined.

**Results:**

A total of 274 *P. falciparum* and 70 *P. vivax* cases were enrolled. The day-42 PCR-corrected ACPR for *P. falciparum* was 98.1% (104/106) for AL and 100% (135/135) for DHA-PPQ. The day-42 PCR-corrected ACPR for *P. vivax* was 79.0% (15/19) for AL and 92.3% (36/39) for DHA-PPQ. Day 3 parasite clearance of *P. falciparum* was 99.2% with AL and 100% with DHA-PPQ. In vitro testing of 96 samples revealed low susceptibility to chloroquine (34% of samples above IC_50_ threshold) but not to lumefantrine (0%). Molecular markers assessed in a sub-set of the study population indicated high rates of chloroquine resistance in *P. falciparum* (*pfcrt* SVMNT: 94.2%, n = 104) and in *P. vivax* (*pvmdr1* Y976F: 64.8%, n = 54).

**Conclusions:**

AL and DHA-PPQ were efficacious as first- and second-line treatments for uncomplicated malaria in PNG. Continued in vivo efficacy monitoring is warranted considering the threat of resistance to artemisinin and partner drugs in the region and scale-up of artemisinin-based combination therapy in PNG.

**Electronic supplementary material:**

The online version of this article (10.1186/s12936-018-2494-z) contains supplementary material, which is available to authorized users.

## Background

The widespread resistance to commonly used 4-aminoquinolines continues to impede malaria control strategies in malaria-endemic countries. For decades the anti-malarial drugs chloroquine and sulfadoxine-pyrimethamine were highly effective against falciparum malaria. However, due to the emergence of resistant *Plasmodium falciparum* and *Plasmodium vivax* parasite strains [1], the World Health Organization (WHO) now recommends a 3-day course of artemisinin-based combination therapy (ACT), which has a proven high efficacy [2]. By administering a 3-day regimen, the concentration of both drugs is effective enough to kill the erythrocytic stages of the parasite [3]. Moreover, the risk of a spread of strains resistant to artemisinin derivatives is minimized by the partner drug. In line with WHO recommendations, Papua New Guinea (PNG) introduced a new anti-malarial treatment guideline in 2009 with artemether–lumefantrine (AL) and dihydroartemisinin–piperaquine (DHA-PPQ) as the first-and second-line treatments, respectively [4, 5]. A comparative treatment trial conducted in PNG children had found AL to be the most efficacious treatment against *P. falciparum* and DHA-PPQ most efficacious against *P. vivax* [6]. The country-wide roll-out of AL to public health facilities started 2 years after policy adoption, in late 2011 [7].

Unfortunately, there is increasing resistance of parasites to ACT in the Greater Mekong Region [4, 8, 9], a region that is well known for the development of anti-malarial drug resistance. Recently, alarming rates of resistance to DHA-PPQ have been reported in Cambodia [10]. In order to protect the efficacy of ACT drug efficacy, monitoring is considered an essential aspect of malaria control programmes in all endemic countries [11, 12].

A therapeutic efficacy study was conducted in PNG with the primary objective of assessing the in vivo efficacy of AL and DHA-PPQ after their introduction. Secondary objectives included measuring the in vitro efficacy (IC_50_) of a range of anti-malarial drugs used in PNG and investigating the prevalence of molecular markers associated with in vivo failures.

## Methods

### Study sites

This study was conducted in 2 sites in PNG (Maprik, East Sepik Province, and Alotau, Milne Bay Province) from 2012 to 2014 following the standard WHO protocol for surveillance of anti-malarial drug efficacy [13]. In Maprik, patients were recruited from Ilahita Health Centre and the Sunuhu and Malahum aid posts. In Alotau, patients were recruited from Gurney Health Centre, Alotau General Hospital Children’s Outpatient Department, Goilanai Urban Clinic, Hagita Estate Health Centre, and from the Gabugabuna and Naura Aid Posts.

### Patient enrolment and follow-up

Patients aged ≥ 6 months presenting with fever (≥ 37.5 °C) or a history of fever in the previous 72 h at one of the study health facilities were initially screened and enrolled provided they had: (i) weight ≥ 5.0 kg; (ii) Hb ≥ 5.0 g/dL; (iii) easy access to the study facility to enable follow-up; (iv) positive malaria rapid diagnostic test (RDT; CareStart Malaria Combo, Access Bio, USA); and, (v) microscopy-confirmed *P. falciparum* (≥ 1000 parasites/μL) or *P. vivax* (≥ 250 parasites/μL) mono-infection. Patients with signs of severe malaria and other exclusion criteria were referred to health facility staff for clinical management according to routine practice. Patients were enrolled and allocated to either the AL or DHA-PPQ treatment arm. Due to a delay in the arrival of the second-line drug (DHA-PPQ) in the country, the AL treatment arm was started first followed by the DHA-PPQ arm a year later. Patients were initially enrolled on a provisional basis based on their RDT result and treated with AL or DHA-PPQ. After confirmatory diagnosis by light microscopy, only patients meeting the above inclusion criteria were retained in the study. RDT-positive cases that had parasite counts below the required threshold or mixed species infections were excluded while a complete treatment course was provided.

Active follow-up was performed by study nurses on days 1, 2, 3, 7, 14, 28, and 42. During enrolment and follow-up visits, clinical and physical examinations were performed and the findings were recorded in case report forms. Blood slides and filter paper samples were collected in all visits while haemoglobin levels were measured on days 0, 7, 14, 28, and 42 using a hand-held Hemocue device (Hb201+, Hemocue, Sweden).

### Drugs administration

Patients enrolled into the AL arm were treated with 6 doses of Coartem^®^ tablets (Novartis Pharma, Switzerland) containing 20 mg of artemether and 120 mg of lumefantrine over 3 days, administered with 250 mL of milk. Doses were allocated by weight group: 5.0–14.9 kg 1 tablet; 15.0–24.9 kg 2 tablets; 25.0–34.9 kg 3 tablets; and ≥ 35.0 kg 4 tablets. All doses were administered under direct supervision by a study nurse. For patients enrolled into the DHA-PPQ arm, Eurartesim^®^ film-coated tablets (Sigma-tau Pharma Limited, Rome, Italy) containing 40 mg of DHA and 320 mg of PPQ were administered with water under supervision once daily for 3 days. Doses were allocated according to the following weight groups: 7.0 to < 13.0 kg ½ tablet; 13.0 to < 24.0 kg 1 tablet; 24.0 to < 36.0 kg 2 tablets; 36.0 to < 75.0 kg 3 tablets; 75.0–100.0 kg 4 tablets. The dosage schedules were in accordance with the National Malaria Treatment Protocol [5]. Participants who vomited within 30 min of treatment administration were re-administered the dose from a spare blister. Participants who were parasitaemic during a follow-up visit were excluded and referred to the study health facility for treatment, while the study team ensured that all participants were eventually completely cured.

### Laboratory procedures

Light microscopic diagnosis was conducted independently by 2 WHO-certified Level 1 to Level 3 microscopists at the Papua New Guinea Institute of Medical Research (PNGIMR) in Madang. In case of discordant results, slides were examined by a third senior microscopist (WHO Level 1 certified). A minimum of 200 thick film fields were examined before a slide was declared negative and a patient was considered aparasitaemic if no parasites were found by two microscopists. The number of parasites was counted up to 200 white blood cells (parasite count > 100/field) or 500 white blood cells (parasite count < 100/field). Parasite counts for each species were converted to the number of parasites per μL of blood assuming 8000 white blood cells per μL [11].

Polymerase chain reaction (PCR) assays were used to distinguish recrudescence from re-infection cases using *Pfmsp2* for *P. falciparum* and *Pvmsp1* for *P. vivax* [14–17]. Length polymorphisms of the fragments of *Pfmsp2*, *Pvmsp1F3* and *Pvms16* were determined using capillary electrophoresis at Macrogen (South Korea). The data obtained from GeneScan (Thermo Fisher Scientific, Waltham, MA, USA) were analysed using GeneMarker, version 2.4.0 (Soft Genetics, State College, PA, USA). The method used for *P. vivax* allows discrimination between strains in post-treatment recurrent infections in a way analogous to that established for falciparum malaria. Although *P. vivax* genotyping cannot differentiate between a recrudescent infection and a relapse with the same genotype, based on previous observations, most relapses are genetically distinct from the primary infections [14, 15, 17].

A ligase detection reaction-fluorescent microsphere assay (LDR-FMA) method, which has been described in detail elsewhere [14, 16, 17], was used for genotyping anti-malarial resistance markers. This study focussed on the presence of single point mutations for the *P. falciparum* chloroquine-resistance transporter gene (*pfcrt*K76T) and the *P. falciparum* multi-drug resistance-1 (*pfmdr*-*1*N86Y, Y184F, S1034C, N1042D & D1246Y) as well as the *P. vivax* multi-drug resistance-1 (*pvmdr*-*1*Y976F) genes. PfK13 for artemisinin and Pfplasmepsin 2/3 for piperaquine resistance were not assessed as this study was carried out before the discovery of these markers.

*Plasmodium falciparum* cultures were maintained using a modified candle-jar technique based on the Trager & Jensen method for culture of *P. falciparum* parasites [18]. The anti-malarial drugs used in this assay were chloroquine, lumefantrine, naphthoquine, piperaquine, and pyronaridine. The plates were set up as previously described [19], with modifications.

### Study outcomes

The treatment outcomes were categorized according to WHO guidelines [13] as early treatment failure (ETF): having danger signs or complicated malaria with evidence of persistent parasitaemia and fever (≥ 37.5 °C) on the first 3 days of treatment; late clinical failure (LCF), defined as the detection of recurrent parasitaemia with fever (≥ 37.5 °C) between day 4 and day 28 (day 42) without meeting any criteria of ETF; late parasitological failure (LPF): the detection of parasitaemia between day 7 and day 28 (day 42) but with no signs of symptomatic malaria; and, adequate clinical and parasitological response (ACPR) where the treatment is completed successfully with no confirmed re-infection on day 28 (day 42) and without meeting previous criteria of ETF, LCF or LPF. The overall treatment failure was considered as the sum of the ETF, LCF and LPF. Fever clearance was defined as proportion of patients whose body temperature decreased below 37.3 °C after drug intake on days 1, 2 and 3. Parasite clearance was defined as the proportion of patients with a negative blood slide after drug intake on days 1, 2 and 3. IC_50_ values are the concentration of a particular drug at which 50% of the parasites is inhibited in vitro. The prevalence of molecular markers was calculated as the frequency of any of the single nucleotide polymorphisms (SNPs) within the key resistance molecular markers (*pfcrt, pfmdr1* and *pvmdr1*).

### Statistical methods

The sample size calculation for this study was based on an expected 5% failure rate of *P. falciparum,* a 95% level of confidence and 5% precision. An additional 20% was added to account for patients who would be lost during follow-up, violate protocol or voluntarily withdraw, resulting in a total sample size of 88 patients per arm per site, allowing site-specific efficacy estimation. Due to the unpredictability of the incidence of *P. vivax* mono-infections, no formal sample size was performed for including *P. vivax* cases. Instead, the study would recruit all patients with *P. vivax* up until the total *P. falciparum* sample size was reached.

Case report forms were entered using REDCap (Research Electronic Data Capture; http://project-redcap.org/). Analysis of study outcomes was based on a per-protocol analysis of pooled patient data from both study sites, which excluded cases with a *Plasmodium* species other than *P. falciparum* or *P. vivax*, protocol violations, and patients lost to follow-up. Statistical analysis was carried out using STATA 11.0 (StataCorp, Texas, USA). Statistically significant differences in rates of treatment response outcomes, both for PCR-corrected and PCR-uncorrected, as well as in baseline characteristics were assessed using Chi square tests and non-parametric tests for not normally distributed data. Fever and parasite clearance rates were compared using Fisher’s exact test. Fever was defined as axillary temperature of ≥ 37.5 °C for enrolment and a 37.3 °C cut-off was used to define fever clearance.

## Results

### Baseline characteristics

**Fig. 1:**
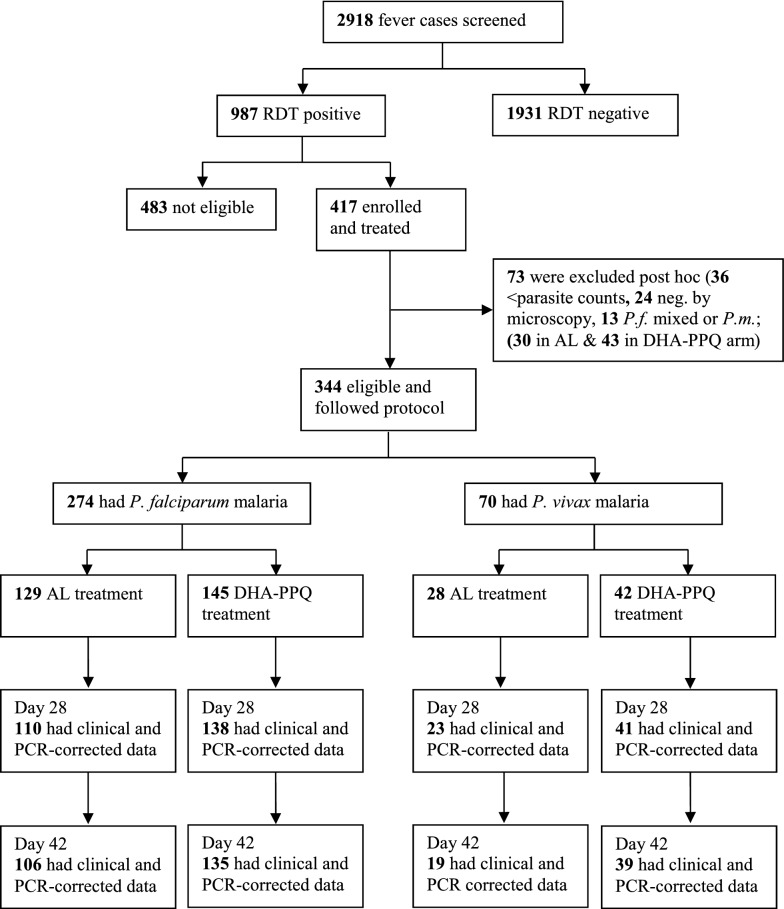
Screening, enrolment, treatment, and follow-up of study patients from Maprik and Alotau, Papua New Guinea, June 2012–September 2014

A total of 2918 patients with fever (≥ 37.5 °C), or a recent history of fever, were screened at the health facilities in Maprik and Alotau between June 2012 and September 2014 (Fig. 1). Of these, 33.8% (987/2918) had a positive RDT and 42.2% (417/987) were initially enrolled on a provisional basis and sequentially allocated to either the AL or DHA-PPQ study arm. Out of the provisionally enrolled patients, 17.5% (73/417) were excluded post hoc, including 49.3% (36/73) due to too low parasite counts, 32.9% (24/73) were negative by microscopy, while 17.8% (13/73) were mixed infections or *Plasmodium malariae*.

In total, 344 patients (age range 0.5–64 years) were finally enrolled and followed-up according to the study protocol (171 in Alotau and 173 in Maprik). Out of the *P. falciparum* cases in the AL arm, 19 were excluded between day 0 and 28 (7 lost to follow-up, 11 protocol violations, 1 voluntary withdrawal) and 4 between day 28 and 42 (2 protocol violations, 2 lost to follow-up). Of the *P. falciparum* cases in the DHA-PPQ arm, 7 were excluded between day 0 and 28 (3 protocol violations, 3 lost to follow-up, 1 voluntary withdrawal) and 3 were lost to follow-up between day 28 and 42. Of the *P. vivax* cases in the AL arm, 5 were excluded between day 0 and 28 (1 protocol violation, 4 lost to follow-up) and 4 between day 28 and 42 (3 lost to follow-up, 1 protocol violation). Of the *P. vivax* cases in the DHA-PPQ arm, 1 was excluded due to protocol violation between day 0 and 28, while 2 were lost to follow-up between day 28 and 42. A comparison of the two study populations is provided in Additional file 1: Table S1. On average, patients in Maprik were younger (mean age 5.6 years vs 7.9 in Alotau, P < 0.001), lighter (mean weight 16.1 kg vs 20.7 kg, P < 0.001), had a higher prevalence of splenomegaly (49.7 vs 28.1%, P = 0.001) and a lower mean haemoglobin concentration (8.6 g/dL vs 10.5 g/dL, P < 0.001).

Overall, 79.7% (274/344) of all enrolled patients were infected with *P. falciparum* and 20.3% (70/344) with *P. vivax*. Of the patients with falciparum malaria, 47.1% (129/274) were treated with AL while 52.9% (145/274) received DHA-PPQ. Of those treated with AL, 85.3% (110/129) and 82.2% (106/129) had clinical and PCR-corrected data on days 28 and 42, respectively. Of the DHA-PPQ-treated patients, 95.2% (138/145) and 93.1% (135/145) had clinical and PCR-corrected data on days 28 and 42, respectively. Of the patients with vivax malaria, 40% (28/70) were treated with AL and 60% (42/70) with DHA-PPQ. Of those treated with AL, 82.1% (23/28) and 67.9% (19/28) had clinical and PCR-corrected data on days 28 and 42, respectively, while 97.6% (41/42) and 92.9% (39/42) of DHA-PPQ-treated patients had the respective data available (Fig. 1).

Patients enrolled into the AL and DHA-PPQ treatment arms were comparable with regard to most baseline demographic and anthropometric characteristics (gender, weight, mid-upper-arm circumference, Hb, parasite density, enlarged spleen; Table 1). However, there was a significant baseline difference (P = 0.009) in body temperature between the AL and DHA-PPQ-treated patients with vivax malaria. In addition, the median parasite density in patients with falciparum malaria was significantly higher in the DHA-PPQ arm compared to the AL arm (P < 0.001).

**Table 1:** Baseline characteristics of patients according to treatment arms with *Plasmodium falciparum* and *Plasmodium vivax* infections

Characteristics	*P. falciparum* (N = 274)			*P. vivax* (N = 70)		
	AL	DHA-PPQ	*P*-value	AL	DHA-PPQ	*P*-value
Males (%)	48	52	0.74	40	60	> 0.99
Mean age (years)	6.5	8.2	0.04	4.5	4.0	0.53
Mean weight (kg)	18.7	19.9	0.33	15.2	14.0	0.53
Mean MUAC (cm)	17.2	17.5	0.48	16.5	16.5	> 0.95
Parasite density/µL						
Median	13,639	26718	< 0.0001	2835	6302	0.07
Range	1058–26,373	1133–261,992		831–157,339	305–86,666	
Mean temperature (°C)	37.8	37.5	0.08	37.7	36.9	0.009
Enlarge spleen (%)	48.7	51.3	0.65	47.4	52.6	0.59
Mean Hb (g/dL)	9.5	9.5	>0.95	9.7	9.5	0.58

*AL* artemether–lumefantrine, *DHA-PPQ* dihydroartemisinin–piperaquine

### Drug efficacy

Nine of the 274 patients infected with *P. falciparum* at enrolment (N = 274) experienced a treatment failure by day 42. Of these, 8 were classified as LPF (3 in the DHA-PPQ arm and 6 in the AL arm) while 1 was a LCF in the AL arm (Table 2). After PCR correction for re-infection, the day-28 ACPR was 100% in both the AL (110/110) and DHA-PPQ (138/138) arms (Table 3). At day 42 it was 98.1% (104/106) for AL and 100% (135/135) for DHA-PPQ (P = 0.11).

**Table 2:** Per-protocol secondary endpoint analysis of treatment responses in cases with *Plasmodium falciparum* or *Plasmodium vivax* for PCR-uncorrected malaria

	AL	DHA-PPQ	Total	*P*-value
*P. falciparum* assessed at day 28, n	110	138	248	
Adequate clinical and parasitological response, n (%) [95% CI]	108 (98.2) [92.9–99.7]	138 (100) [96.6–100]	246 (99.2)	− 0.28
Early treatment failure, %	0	0	0	–
Late clinical failure, %	0.9	0	0.4	–
Late parasitological failure, %	0.9	0	0.4	–
*P. falciparum* assessed at day 42, n	106	135	241	
Adequate clinical and parasitological response, n (%) [95% CI]	102 (96.2) [90.07–98.8]	132 (97.8) [93.2–99.4]	234 (97.1)	0.48
Early treatment failure, %	0	0	0	–
Late clinical failure, %	0	0	0	–
Late parasitological failure, %	3.8	2.2	2.9	–
*P. vivax* assessed day 28, n	23	41	64	
Adequate clinical and parasitological response, n (%) [95% CI]	20 (87.0) [65.3–96.6]	41 (100) [89.3–100]	61 (95.3)	0.06
Early treatment failure, %	0	0	0	–
Late clinical failure, %	4.3	0	1.6	–
Late parasitological failure, %	8.7	0	3.1	–
*P. vivax* assessed day 42, n	19	39	58	
Adequate clinical and parasitological response, n (%) [95% CI]	13 (68.4) [43.5–86.4]	34 (87.2) [71.8–95.2]	47 (81.03)	0.23
Early treatment failure, %	0	0	0	–
Late clinical failure, %	10.5	5.1	6.9	–
Late parasitological failure, %	21.1	7.7	5.3	–

**Table 3:** Per-protocol primary endpoint analysis of treatment responses in cases with *Plasmodium falciparum* or *Plasmodium. vivax* for PCR-corrected malaria

	AL	DHA-PPQ	Total	*P*-value
*P. falciparum* assessed at day 28, n	110	138	248	
Adequate clinical and parasitological response, n (%) [95% CI]	110 (100) [95.8–100]	138 (100) [96.6–100]	248 (100)	–
Early treatment failure, %	0	0	0	–
Late clinical failure, %	0	0	0	–
Late parasitological failure, %	0	0	0	–
*P. falciparum* assessed at day 42, n	106	135	241	
Adequate clinical and parasitological response, n (%) [95% CI]	104 (98.1) [92–99.7]	135 (100) [96.6–100]	239 (99.2)	− 0.11
Early treatment failure, %	0	0	0	–
Late clinical failure, %	0	0	0	–
Late parasitological failure, %	1.9	0	0.8	–
*P. vivax* assessed day 28, n	23	41	64	–
Adequate clinical and parasitological response, n (%) [95% CI]	22 (95.7) [76.03–99.8]	41 (100) [89.3–100]	63 (98.4)	0.18
Early treatment failure, %	0	0	0	–
Late clinical failure, %	0	0	0	–
Late parasitological failure, %	4.3	0	1.6	–
*P. vivax* assessed day 42, n	19	39	58	–
Adequate clinical and parasitological response, n (%) [95% CI]	15 (78.9) [53.9–93.03]	36 (92.3) [78.03–98.0]	51 (87.9)	0.14
Early treatment failure, %	0	0	0	–
Late clinical failure, %	5.3	0	1.7	–
Late parasitological failure, %	15.8	7.7	10.3	–

Fourteen of the 70 patients infected with *P. vivax* at enrolment had a treatment failure by day 42 (9 in the AL arm and 5 in the DHA-PPQ arm; Table 2), most with LPF. After PCR correction for re-infection 7 remained positive. The PCR-corrected ACPR at day 28 was 95.7% (22/23) for AL, all LPF, and 100% (41/41) for DHA-PPQ (P = 0.18). At day 42, PCR-corrected ACPR was 78.9% (15/19) for AL and 92.3% (36/39) for DHA-PPQ (P = 0.14) (Table 3). Day-28 PCR-uncorrected ACPR was 87% (20/23) and 100% (41/41) for AL and DHA.PPQ, respectively (P = 0.06), while at day 42 it was 68.4% (13/19) for AL and 87.2% (34/39) for DHA-PPQ (P = 0.23; Table 2).

### Fever and parasite clearance

**Fig. 2:**
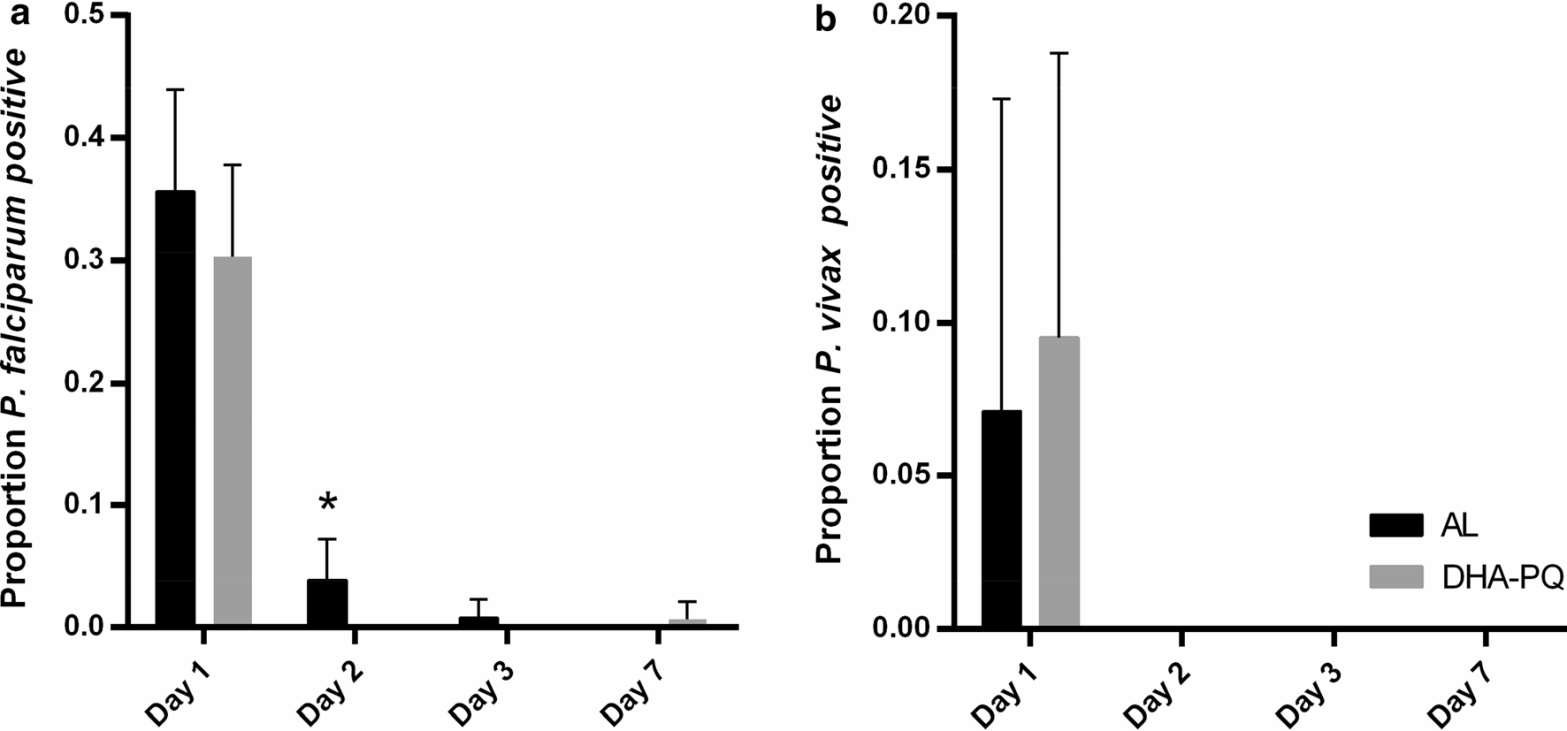
Graphic representation parasite clearance (as in Table 4B).*Indicates a statistically significant difference between treatment groups

Fevers were cleared rapidly with the vast majority of patients being parasite free by day 3 but a significantly higher proportion of *P. falciparum* patients was still febrile on day 1 after treatment with AL as compared to DHA-PPQ (odds ratio = 1.88, Fisher’s Exact Test P = 0.008). However, all fevers in both *P. falciparum*- and *P. vivax*-infected patients in the AL arm had cleared by day 3. One patient with *P. vivax* in the DHA-PPQ arm presented with fever at every time point post treatment (day 0–day 7). One patient with *P. falciparum* in the DHA-PPQ arm had fever on day 2 and then on day 7. However, all these fevers were most likely not associated with the parasite infection as all parasites were cleared in these patients and there were no fevers on day 1 (Table 4, Fig. 2).

**Table 4:** Proportion of patients with no detectable parasites grouped by malaria species and treatment arm

	*P. falciparum*			*P. vivax*		
	AL	DHA-PPQ	P-value*	AL	DHA-PPQ	P-value*
A) Fever clearance**						
Day 1	88.2 (60/68)	100 (64/64)	0.008	92.9 (13/14)	90.1 (10/11)	1.00
Day 2	95.6 (65/68)	98.4 (63/64)	0.62	92.9 (13/14)	90.1 (10/11)	1.00
Day 3	100 (68/68)	96.9 (62/64)	0.24	100 (14/14)	90.1 (10/11)	0.46
Day 7	100 (68/68)	98.4 (63/64)	0.49	100 (14/14)	90.1 (10/11)	0.46
B) Parasite clearance						
Day 1	64.3 (83/129)	70 (101/145)	0.7	92.9 (26/28)	90.5 (38/42)	1.00
Day 2	96.1 (124/129)	100 (142/142)	0.93	100 (28/28)	100 (42/42)	1.00
Day 3	99.2 (128/129)	100 (143/143)	1.00	100 (28/28)	100 (42/42)	1.00
Day 7	100 (129/129)	99 (141/142)	1.00	100 (28/28)	100 (42/42)	1.00

Values are presented as % (n/N)

* Fisher’s exact test

** Fever clearance was defined as axillary temperature < 37.3 °C after two consecutive events with ≥ 37.3 °C starting from day 0

### Haemoglobin

In the AL treatment arm, the mean Hb concentration increased from 9.6 g/dL in patients with *P. falciparum* and 9.7 g/dL in patients with *P. vivax* on day 0 to 10.7 days/dL and 10.2 days/dL on day 42, respectively. Hb levels in *P. vivax* patients appeared to increase faster than in *P. falciparum* patients (Additional file 2: Figure S1). In the DHA-PPQ arm, a decrease in mean Hb was observed on day 7 (8.8 g/dL in *P. falciparum*, 9.2 g/dL in *P. vivax*), followed by an increase on subsequent days. In all groups, day 42 mean Hb concentration was higher than on day 0 and no participant had developed severe anaemia (< 8 g/dL) over the course of the treatment.

### Adverse events

Both treatments were generally well tolerated. The overall frequency of adverse events was 56.1% (88/157) in the AL arm and 60.4% (113/187) in the DHA-PPQ arm (P = 0.443) (Table 5). The most frequent adverse events were cough (38.2% in AL; 38% in DHA-PPQ), running nose (12.1% in AL; 21.9% DHA-PPQ) and headache (10.2% AL and 18.7% DHA-PPQ). Headache and running nose were significantly more frequently reported in patients in the DHA-PPQ arm (P = 0.033 and P = 0.022, respectively). The observed adverse events are common signs related to the underlying disease (malaria) and are likely not a result of the study drugs. There were neither severe adverse events nor any deaths during the study.

**Table 5:** Most frequent adverse events after 28 days of treatment

Adverse events	AL	DHA-PPQ	*P*-value*
	(N = 157)	(N = 187)	
Patients with at least one adverse event	87 (55.4)	112 (59.9)	0.443
Abdominal pain	17 (10.8)	10 (5.3)	0.071
Chills	12 (7.6)	15 (8.0)	1.0
Cough	60 (38.2)	71 (38.0)	1.0
Diarrhoea	11 (7.0)	8 (4.3)	0.345
Headache	16 (10.2)	35 (18.7)	0.033
Vomit	4 (2.5)	6 (3.2)	0.760
Ear pain	3 (1.9)	2 (1.1)	0.663
Running nose	19 (12.1)	41 (21.9)	0.022
Skin rash	1 (0.6)	2 (1.1)	1.0
Shortness of breath	1 (0.6)	2 (1.1)	1.0
Fits	1 (0.6)	2 (1.1)	1.0
Stiff neck	1 (0.6)	1 (0.5)	1.0

Values are presented n (%)

* Fisher’s exact test

### Molecular markers of resistance

Out of a total of 274 *P. falciparum* cases, 104 had a *pfcrt* LDR-FMA confirmed result. The single mutant haplotype (SVMNT) was found in 96 (92.3%) samples, the polyclonal CVMNK/SVMNT mutant haplotype in 2 (1.9%), and the wild type (CVMNK) in 6 (5.8%) cases and 2 (1.9%) exhibited the polyclonal CVMNK/SVMNT mutant haplotype. Out of the two confirmed LPF in *P. falciparum* patients on day 42, one carried the SVMNT mutation. The genotype of the second patient could not be determined. Three cases treated with DHA-PPQ had a SVMNT mutation with 1 exhibiting a chloroquine IC_50_ value of 122 nM. The only polyclonal (CVMNK/SVMNT) isolate that was treated with AL had a chloroquine IC_50_ of 67.5 nM.

For *pvmdr1*, the Y976F mutation was assessed. A total of 54 samples were successfully typed of which 19 (35.2%) were wild type (Y), 26 (48.1%) were mutant (F) and 9 (16.7%) were mixed wild type mutant (YF). Of the 6 *P. vivax* cases that had LPF at day 42 (3 from each treatment arm), 2 were wild type, 2 were mutant, 1 was mixed wild type/mutant and 1 was undetermined. The only LCF with *P. vivax* and treated with AL was a mutant.

### In vitro drug susceptibility

A total of 49 *P. falciparum* parasite isolates from Maprik and 47 from Alotau were collected at enrolment were tested for in vitro sensitivity to chloroquine, lumefantrine, naphthoquine, piperaquine, and pyronaridine. The mean drug concentration required for 50% parasite growth inhibition in vitro (IC_50_) is provided in Table 6. In total, 34% (33/96) of the chloroquine assays had an IC_50_ value above the ≥ 100 nM cut-off, while all lumefantrine had IC_50_ values below their ≥ 150 nM cut-off threshold. No cut-off values for piperaquine and pyronaridine were available in literature.

**Table 6:** *In vitro* drug susceptibility measured in the *Plasmodium falciparum* isolates collected in Maprik and Alotau and comparison with results from study previously conducted in Madang (2011–2013)

Drug	Maprik			Alotau			Maprik vs Alotau	Madang [20]			Maprik vs Madang	Alotau vs Madang	Cut-off (nM)**
	N	Mean	95% CI	N	Mean	95% CI	P-value*	N	Mean	95% CI	P-value*	P-value*	
Chloroquine	49	65.26	50.04–85.1	47	74.00	59.38–92.21	0.59	48	86.76	70.66–106.5	0.09	0.19	≥ 100
Lumefantrine	49	6.04	4.74–7.7	47	7.97	6.46–9.82	0.18	44	1.55	1.14–2.1	< 0.0001	< 0.0001	≥ 150
Naphthoquine	49	7.30	6.1–8.73	47	8.79	7.41–10.42	0.19	47	4.24	3.13–5.76	0.0003	< 0.0001	
Piperaquine	49	18.60	14.27–24.23	47	23.41	17.46–31.38	0.15	47	21.02	16.96–26.04	0.44	0.31	
Pyronaridine	49	9.33	7.02–12.4	47	10.02	7.92–12.61	0.98	45	7.97	5.99–10.61	0.11	0.19	

Values are presented as geometric mean IC_50_ and 95% confidence interval (CI)

* Mann–Whitney U test

** Cut off values referring to in vivo resistance suggested in the literature

There were no significant differences observed between the two study sites in the geometric mean IC_50_ to any anti-malarials tested (P for all > 0.05). For the two PCR confirmed LPF with *P. falciparum* (day 42) in the AL treatment arm, no in vitro assays were performed.

A further comparison was undertaken between in vitro drug susceptibility data collected in Madang between 2011 and 2013 [20] and those generated in the present study. The comparison is also shown in Table 6. The assays were carried out under comparable conditions using the same study drugs. Geometric mean IC_50_ values for naphthoquine and lumefantrine differed significantly. Both drugs seemingly exhibited a lower susceptibility in the present study as compared to the previous trial in Madang.

## Discussion

This therapeutic efficacy study conducted across all age groups in two sites of PNG confirms high efficacy of the current first- and second-line anti-malarial treatments, AL and DHA-PPQ, for uncomplicated malaria. No recrudescence was found within 42 days in patients with falciparum malaria treated with DHA-PPQ but 2 (1.9%) patients treated with AL experienced LPF. ACPR in AL-treated patients was comparable to two other studies carried out in children aged 0.5–5 years in Madang and East Sepik provinces (Karunajeewa et al. day 42 ACPR 95.2% [6]; Laman et al. day 42 ACPR 97.8% [11]. The two recrudescent infections observed in the AL arm on day 42 underline the importance of continued monitoring of the efficacy of AL in the context of increasing use of the regimen in public facilities and persisting availability of monotherapy [21]. ACPR to DHA-PPQ was higher in this study (day 42: 97.8%, 95% CI 93.2–99.4) than in the previous trial conducted by Karunajeewa et al. in 2005–2007 (day 42: 88%, 95% CI 80–93.6) [6]. The 95% confidence intervals of the ACPR of both drugs remain well above the 90% minimum efficacy threshold defined by WHO [2].

The interpretation of the efficacy against *P. vivax* cases is compromised mainly by the inherent difficulty of unambiguously distinguishing recrudescences from relapses and re-infections. PCR-uncorrected ACPR on day 42 was 68.4% (95% CI 43.5–86.4) in patients treated with AL and 87.2% (95% CI 71.8–95.2) in patients treated with DHA-PPQ. ACPR was higher in this study than previously reported for AL (30.3%, 95% CI 15.6–48.7) and DHA-PPQ (69.4%, 95% CI 51.9–83.7) in the study by Karunajeewa et al. [6]. Of all *P. vivax* cases with recurrent parasitaemia on day 42, 33.3% in the AL group and 40% in the DHA-PPQ group were identified as infections with new genotypes suggestive of a re-infection or relapse rather than recrudescence. The resulting PCR-corrected ACPR of both treatment regimens in this study remained above 90% on day 28 and on day 42 in the case of DHA-PPQ; however, confidence intervals spanned across the 90% efficacy threshold generally applied for *P. falciparum*. As genotyping cannot differentiate between true recrudescence and relapses from hypnozoites with the same genotype, the PCR-corrected ACPR values for *P. vivax* need to be interpreted with caution. At day 28, there were 4.3% treatment failures seen in the AL group but none in DHA-PPQ. While these results may be indicative of a higher efficacy of DHA-PPA against *P. vivax*, as suggested in a previous study to be likely due to the longer half-life of the partner drug PPQ [6] this was not statistically significant in the present study as it was not powered for such comparison.

This study further assessed parasite and fever clearance time over the first 7 days in order to detect any delayed treatment response. Day 3 parasite clearance after treatment with an ACT has been suggested by WHO as a proxy indicator for artemisinin resistance of *P. falciparum*. An increased parasite clearance time with ≥ 10% of cases with detectable *P. falciparum* parasites on day 3 is the current working definition of suspected artemisinin resistance [9]. This study found high parasite and fever clearance rates in patients infected with *P. falciparum* or *P. vivax* in both treatment groups, confirming the general observation that both, AL and DHA-PPQ are still highly efficacious treatments in PNG.

The drop in Hb concentration observed in *P. falciparum* patients treated with DHA-PPQ may be explained by the higher average parasite density in this study group (mean density in *P. falciparum* patients AL 19,298 parasites/μL vs DHA-PPQ 41,282 parasites/μL; P < 0.001; mean density in *P. vivax* patients AL 10,229 parasites/μL vs DHA-PPQ 9356 parasites/μL; P = 0.87). On the other hand, a decrease in Hb in patients treated with DHA-PPQ had previously been observed in other studies and further investigations may hence be warranted [6].

There were no significant differences in in vitro efficacy of the tested drugs between the study sites (Alotau and Maprik). A recent study using the same assay conducted in Madang [20] found similar IC_50_ values. When comparing the results from this study with a previous study carried out in Madang 5 years ago [22], differences were observed in IC_50_ in Madang and Alotau or Maprik, respectively, were: chloroquine (167 nM vs 69 nM), piperaquine (27.7 nM vs 21 nM), and lumefantrine (1.55 nM vs 6.92 nM). Further studies are required to determine if these differences are due to the use of a different assay (Sybr green in this study vs pLDH in the previous study). In addition, the lower IC_50_ values of chloroquine seen in this study could possibly be an early indication of decreased parasite resistance pressure due to the shelving of chloroquine in PNG over the past 6 years.

The mutations in the *pfcrt* gene however remained near fixation (> 95% of samples had mutant *pfcrt*). Mutations in the *pfmdr* gene were also widespread with 68% of isolates carrying the N86Y mutation with other mutations in *pfmdr* being rare or absent. These results are in accordance with the prevalence of molecular markers of resistance as shown in earlier studies [20, 22] and indicate that chloroquine resistance is likely to still be widespread in PNG. The Y976F mutation has previously been associated with *P. vivax* chloroquine resistance in Melanesia [23]. The higher prevalence of this mutation thus indicated that *P. vivax* chloroquine resistance is likely to be common in PNG, thus justifying the use of ACT as first-line treatments against both *P. falciparum* and *P. vivax*.

Implementing this study was faced with operational challenges as only a few centres in PNG are equipped for conducting therapeutic efficacy studies. Furthermore, the start of enrolment coincided with a large-scale free distribution of long-lasting insecticidal nets in the study sites which led to a reduction in the number of malaria cases presenting to the health facilities [24]. A considerable number of malaria-positive patients had to be excluded from this study due to low parasite counts making it difficult to recruit a sufficient number of patients for the efficacy study. The final sample size reached did not allow an independent assessment by site but was sufficient for a pooled analysis. Routine in vivo treatment efficacy monitoring hence remains challenging in PNG. Over the last 2 years molecular markers of resistance to artemisinin (i.e., SNPs in the *kelch 13* gene [25] and piperaquine (amplification of plasmepsin 2/3) have been validated and an increase in pfmdr1 86Y wild type alleles have been implicated in increased resistance to AL [26]. These molecular markers thus provide an easier and cheaper alternative that allows continuous monitoring of resistance across numerous locations in PNG.

## Conclusions

The results from this study have shown that AL and DHA-PPQ remain efficacious for the treatment of uncomplicated falciparum and vivax malaria in PNG. Based on the day-3 parasite clearance rate, there is no evidence of artemisinin resistance in the two study sites. Continued monitoring of anti-malarial drug efficacy is warranted considering increasing use of ACT in routine clinical practice, the persistence of artemisinin monotherapy in the system and the presence of artemisinin resistance in neighbouring regions. Regular molecular monitoring of resistance markers may be a more cost-effective alternative to the considerable investments necessary to conduct therapeutic efficacy studies in new or additional study sites in PNG.

## Additional files

**Additional file 1: Table S1.** Study population and key outcomes by study site.

**Additional file 2: Figure S1.** Haemoglobin concentration.

## Abbreviations

AL: artemether–lumefantrine
DHA-PPQ: dihydroartemisinin–piperaquine
ACPR: adequate clinical and parasitological response
ETF: early treatment failure
IRB: Institutional Review Board
LCF: late clinical failure
LPF: late parasitological failure
LDR-FMA: ligase detection reaction-fluorescent microsphere assay
MRAC: Medical Research Advisory Committee
PCR: polymerase chain reaction
Pfmsp2: *Plasmodium falciparum* merozoite surface protein 2
*Pfcrt*: *Plasmodium falciparum* chloroquine transporter
*Pvmdr*-*1*: *Plasmodium vivax* multiple drug resistance 1
PNG: Papua New Guinea
PNGIMR: Papua New Guinea Institute of Medical Research
RDT: rapid diagnostic test
WHO: World Health Organization

## References

[1] WHO (2017). World malaria report 2017.

[2] WHO (2015). Guidelines for the treatment of malaria.

[3] Guerin PJ, Bates SJ, Sibley CH (2009). Global resistance surveillance: ensuring antimalarial efficacy in the future. Curr Opin Infect Dis..

[4] Dondorp AM, Nosten F, Yi P, Das D, Phyo AP, Tarning J (2009). Artemisinin resistance in *Plasmodium falciparu*m malaria. N Engl J Med.

[5] PNG Department of Health (2009). National malaria treatment policy.

[6] Karunajeewa HA, Mueller I, Senn M, Lin E, Law I, Gomorrai PS (2008). A trial of combination antimalarial therapies in children from Papua New Guinea. N Engl J Med.

[7] Pulford J, Kurumop SF, Ura Y, Siba PM, Mueller I, Hetzel MW (2013). Malaria case management in Papua New Guinea following the introduction of a revised treatment protocol. Malar J..

[8] Bhumiratana A, Intarapuk A, Sorosjinda-Nunthawarasilp P, Maneekan P, Koyadun S (2013). Border malaria associated with multidrug resistance on Thailand-Myanmar and Thailand-Cambodia borders: transmission dynamic, vulnerability, and surveillance. Biomed Res Int.

[9] Ringwald P, WHO (2017). Artemisinin and artemisinin-based combination therapy resistance. Drug efficacy and response.

[10] Amaratunga C, Lim P, Suon S, Sreng S, Mao S, Sopha C (2016). Dihydroartemisinin–piperaquine resistance in *Plasmodium falciparum* malaria in Cambodia: a multisite prospective cohort study. Lancet Infect Dis..

[11] Laman M, Moore BR, Benjamin JM, Yadi G, Bona C, Warrel J (2014). Artemisinin-naphthoquine versus artemether–lumefantrine for uncomplicated malaria in Papua New Guinean children: an open-label randomized trial. PLoS Med..

[12] WHO (2011). Global plan for artemisinin resistance containment (GPARC).

[13] WHO (2009). Methods for surveillance of antimalarial drug efficzcy.

[14] Koepfli C, Mueller I, Marfurt J, Goroti M, Sie A, Oa O (2009). Evaluation of *Plasmodium vivax* genotyping markers for molecular monitoring in clinical trials. J Infect Dis.

[15] Mueller I, Widmer S, Michel D, Maraga S, McNamara DT, Kiniboro B (2009). High sensitivity detection of Plasmodium species reveals positive correlations between infections of different species, shifts in age distribution and reduced local variation in Papua New Guinea. Malar J..

[16] Carnevale EP, Kouri D, DaRe JT, McNamara DT, Mueller I, Zimmerman PA (2007). A multiplex ligase detection reaction-fluorescent microsphere assay for simultaneous detection of single nucleotide polymorphisms associated with *Plasmodium falciparum* drug resistance. J Clin Microbiol.

[17] Barnadas C, Kent D, Timinao L, Iga J, Gray LR, Siba P (2011). A new high-throughput method for simultaneous detection of drug resistance associated mutations in *Plasmodium vivax dhfr, dhps* and *mdr1* genes. Malar J..

[18] Trager W, Jensen JB (1976). Human malaria parasites in continuous culture. Science.

[19] Bennett TN, Paguio M, Gligorijevic B, Seudieu C, Kosar AD, Davidson E (2004). Novel, rapid, and inexpensive cell-based quantification of antimalarial drug efficacy. Antimicrob Agents Chemother.

[20] Koleala T, Karl S, Laman M, Moore BR, Benjamin J, Barnadas C (2015). Temporal changes in *Plasmodium falciparum* anti-malarial drug sensitivity in vitro and resistance-associated genetic mutations in isolates from Papua New Guinea. Malar J..

[21] Pulford J, Smith I, Mueller I, Siba PM, Hetzel MW (2016). Health worker compliance with a ‘Test And Treat’ malaria case management orotocol in Papua New Guinea. PLoS One.

[22] Wong RP, Lautu D, Tavul L, Hackett SL, Siba P, Karunajeewa HA (2010). In vitro sensitivity of *Plasmodium falciparum* to conventional and novel antimalarial drugs in Papua New Guinea. Trop Med Int Health..

[23] Marfurt J, de Monbrison F, Brega S, Barbollat L, Muller I, Sie A (2008). Molecular markers of in vivo *Plasmodium vivax* resistance to amodiaquine plus sulfadoxine-pyrimethamine: mutations in pvdhfr and pvmdr1. J Infect Dis.

[24] Hetzel MW, Reimer LJ, Gideon G, Koimbu G, Barnadas C, Makita L (2016). Changes in malaria burden and transmission in sentinel sites after the roll-out of long-lasting insecticidal nets in Papua New Guinea. Parasit Vectors..

[25] Ariey F, Witkowski B, Amaratunga C, Beghain J, Langlois AC, Khim N (2014). A molecular marker of artemisinin-resistant *Plasmodium falciparum* malaria. Nature.

[26] Lobo E, de Sousa B, Rosa S, Figueiredo P, Lobo L, Pateira S (2014). Prevalence of *pfmdr1* alleles associated with artemether–lumefantrine tolerance/resistance in Maputo before and after the implementation of artemisinin-based combination therapy. Malar J..

