# Supplementary material for: Efficacy of artemether–lumefantrine and dihydroartemisinin–piperaquine for the treatment of uncomplicated malaria in Papua New Guinea
Source: Malar J. 2018 Oct 5;17:350. doi: 10.1186/s12936-018-2494-z (PMC6173938; doi:10.1186/s12936-018-2494-z)
Supplement: Supplementary file 1 — Additional file 1: Table S1. Study population and key outcomes by study site. [file 12936_2018_2494_MOESM1_ESM.docx]

**Additional file 1: Table S1:** Study population and key outcomes by study site

| **Characteristics** | **Alotau (N=171)** | **Maprik (N=173)** | ***P*-value** |
| --- | --- | --- | --- |
| Males % (n) | 57.9(99) | 52.6(91) | 0.32 |
| Mean age (years)[95%CI] | 7.9[6.7-9.2] | 5.6[5.0-6.1] | <0.001 |
| Mean weight (kg)[95%CI] | 20.7[18.8-22.6] | 16.1[15.0-17.1] | <0.001 |
| Mean MUAC (cm)[95% CI] | 18.4[17.9-19.0] | 16.0[15.7-16.3] | <0.001 |
| *P. falciparum* density*/*µl |  |  |  |
| Median | 14,209 | 20,813 | 0.08 |
| Range | 1058-263,473 | 1133-122,939 |  |
| *P. vivax* density*/*µl |  |  |  |
| Median | 3656 | 4375 | 0.73 |
| Range | 1062-157,339 | 305-86,666 |  |
| Mean temperature (°C)[95% CI] | 37.7[37.5-37.9] | 37.3[37.1-37.5] | 0.004 |
| Enlarge spleen (%) | 25.5 | 49.7 | <0.001 |
| Mean Hb (g/dl)[95% CI] | 10.5[10.2-10.7] | 8.6[8.3-8.9] | <0.001 |
| AL arm allocation %(n/n) | 54.1(85/157) | 45.9(72/157) | 0.13 |
| DHA-PPQ arm allocation % (n/n) | 46.0(86/187) | 54.0(101/187) | - |
| *P. falciparum* ACPR % (n/n) [95% CI] |  |  |  |
| AL, Day 28 | 100(56/56)  [92-100] | 100(54/54)  [91.7-100] | - |
| AL, Day 42 | 100(52/52)  [91.4-100] | 96.3 (52/54)  [86.2-99.4] | 0.16 |
| DHA-PPQ, Day 28 | 100(55/55)  [91.9-100] | 100 (83/83)  [94.5-100] | - |
| DHA-PPQ, Day 42 | 100(54/54)  [91.7-100] | 100 (81/81)  [94.4-100] | - |
| *P. vivax* ACPR*%(n/n) [95% CI] |  |  |  |
| AL, Day 28 | 92.9 (13/14)  [64.2-99.6] | 100 (9/9)  [62.9-100] | 0.41 |
| AL, Day 42 | 90.0 (9/10)  [54.1-99.5] | 66.7 (6/9)  [30.9-91.0] | 0.21 |
| DHA-PPQ, Day 28 | 100 (29/29)  [85.4-100] | 100 (12/12)  [69.9-100] | - |
| DHA-PPQ, Day 42 | 92.9 (26/28)  [75.1-98.8] | 90.9 (10/11)  [57.1-99.5] | 0.84 |
