# Supplementary material for: Efficacy of artemether–lumefantrine and dihydroartemisinin–piperaquine for the treatment of uncomplicated malaria in Papua New Guinea
Source: Malar J. 2018 Oct 5;17:350. doi: 10.1186/s12936-018-2494-z (PMC6173938; doi:10.1186/s12936-018-2494-z)
Supplement: Supplementary file 2 — Additional file 2: Figure S1. Haemoglobin concentration. [file 12936_2018_2494_MOESM2_ESM.docx]

**Additional file 2: Figure S1:** Haemoglobin concentration


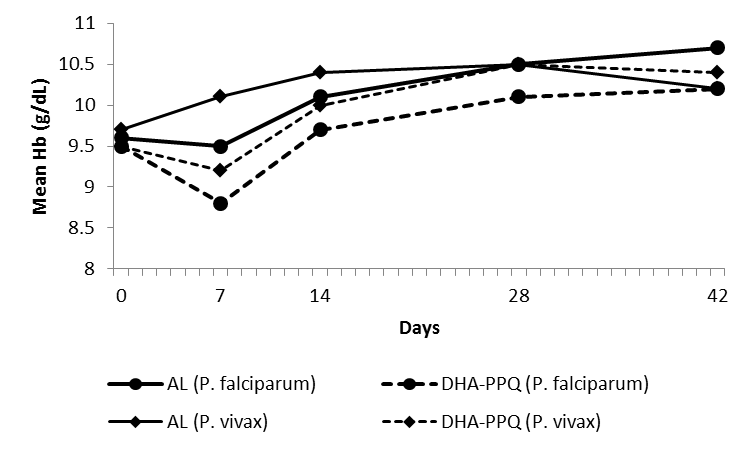


|  | ***P. falciparum*** | | ***P. vivax*** | |
| --- | --- | --- | --- | --- |
| **Day** | **AL** | **DHA-PPQ** | **AL** | **DHA-PPQ** |
| 0 | 9.6 (9.2-10.0) | 9.5 (9.2-9.8) | 9.7 (9.5-10.8) | 9.5 (9.1-10.0) |
| 7 | 9.5 (9.2-9.9) | 8.8 (8.6-9.1) | 10.1 (8.9-10.5) | 9.2 (8.7-9.9) |
| 14 | 10.1 (9.8-10.3) | 9.7 (9.3-10.1) | 10.4 (9.9-10.8) | 10.0 (9.7-10.4) |
| 28 | 10.5 (10.2-10.8) | 10.1 (9.9-10.4) | 10.5 (9.9-11.1) | 10.5 (10.1-10.9) |
| 42 | 10.7 (10.4-11.0) | 10.2 (10.0-10.4) | 10.2 (9.6-10.8) | 10.4 (10.0-10.8) |
